# The Evaluation Criteria in Diagnosing Ischemia with Stress and Rest Myocardial Perfusion Gated SPECT

**DOI:** 10.4274/Mirt.09797

**Published:** 2013-04-05

**Authors:** Cengiz Taşçı

**Affiliations:** 1 Special Gamma Nuclear Medicine Laboratory, Gaziantep, Turkey

**Dear Editor,**

The study named “The role of myocardial perfusion gated SPECT study in women with coronary artery disease” reveals some current and important issues in diagnosing myocardial ischemia in women (1). Because the obesity is the highest and the death from coronary artery disease (CAD) is the second highest in Turkish women among all European countries, diagnosing CAD in women becomes much more important in daily cardiology and nuclear cardiology practice in our country. We have to pay more attention in clarifying the gender-related differences in nuclear cardiology. This study raises awareness about the issue.

On the other hand the evaluation criterion for diagnosing ischemia seems controversial in the study. The question is that: “Do the left ventricular (LV) wall motion abnormality and decreased systolic thickening at the segment with reduced perfusion in gated SPECT study necessarily show true ischemia as it is expressed in the study?

This approach considers that wall motion abnormality and perfusion defects occur exactly at the same time in CAD stages, but as it is known, wall motion abnormalities follow quite a bit later after the perfusion defects appear. Myocardial hypoperfusion is first seen in ischemic cascade, and followed by decrease in metabolic activity, relaxation impairment (diastolic dysfunction), reduction in contractility (systolic dysfunction), global LV dysfunction, ECG changes and angina pectoris, respectively (2). So, the evaluation criterion in the study is true for the advanced stages of ischemia, but not in the beginning of the disease. In the beginning, ischemia may be seen with completely normal wall motion and systolic thickening. So, the approach in the study neglects the ischemic patients with normal cardiac wall motions.

More importantly, because the imaging begins 30-60 minutes after the radiopharmaceutical injection, stress–gated images with Tc-99m MIBI show stress perfusion at the time of injection, but LV function (wall motion, systolic thickening or ejection fraction) at the time of acquisition. Snapper et al. demonstrated that the combination of a perfusion defect with normal wall motion and thickening in the same segment on stress-gated MIBI images was associated with a high positive predictive value (96%) for detecting ischemia. They concluded that the diagnosis of ischemia could be made confidently in patients with normal wall thickening in a segment with perfusion defect. The lack of wall thickening was found less specific, because only 40% of the cases with perfusion defects and wall motion abnormalities showed reversibility on rest perfusion imaging (3,4). Actually, this conclusion is also not always true in daily nuclear cardiology practice, even if there is no soft tissue attenuation. This evaluation accepts that the time course of the resolution of ischemia-induced stunning and LV dysfunction ends immediately after completion of exercise or pharmacological stress. However, there is a lot of evidence demonstrating that the regional wall motion abnormalities persist for variable time periods lasting in seconds, 30 minutes or up to 2-6 hours associated with the severity of stress-induced ischemia. Relatively longer time periods of persistence is particularly due to the presence of repetitive stunning (5). Thus, the functional evaluation in Tc-99m MIBI stress-gated imaging sometimes shows rest situation with no or resolved myocardial wall motion abnormality in slight form of ischemia, and sometimes shows post-stress wall motion abnormalities persisting up to the time of acquisition in more severe forms. So, the term transient ischemic dilation (TID) gains importance defined as the difference between stress and rest end-systolic and end-diastolic volumes. The volumes are greater in post-stress imaging than those in rest imaging in patients with TID. Additionally, lower LV ejection fraction (LVEF) in stress imaging than in rest imaging indicates prolonged post-ischemic stunning and worse prognosis (5). Because of these reasons, LV functions found in rest echocardiography do not always correlate with the findings on post-stress gated SPECT imaging even when neglecting the problems originated from the technical differences.

Finally, the criterion mentioned in the study could be entirely true and supporting if all the subjects had been shown to have reversible defect (ischemia), but some patients are reported to have myocardial infarction. So the criterion might include myocardial scar in patients with fix defect (particularly when the presence of metabolic activation on the defect related with hibernation was excluded). In the routine evaluation of gated SPECT studies, wall motion abnormality and/or systolic thickening at the same segment with fixed defect primarily indicates myocardial scar. So, the inclusion criterion that the authors used in the study constitutes a great limitation for drawing true conclusions about ischemia and/or infarct without making some comparisons between post-stress and rest end-systolic and end-diastolic volumes or measuring LVEF differences together with the wall motion abnormalities.

The evaluation criteria to differentiate ischemia, infarct and soft tissue attenuation artifacts in stress-rest gated SPECT imaging may be simply summarized as below:

1. Normal perfusion with no wall motion abnormality or TID: Normal

2. Normal perfusion with wall motion abnormality or TID: Stunning (and/or multivessel disease?).

3. Reversible ischemia with no wall motion abnormality, TID or reduced LVEF in stress-gated images: Ischemia.

4. Reversible ischemia with wall motion abnormality, TID or reduced LVEF in stress-gated images: Severe ischemia with increased cardiac event rate.

5. Fixed defect with normal wall motion or wall thickening: Soft tissue attenuation artifact.

6. Fixed defect with wall motion or wall thickening abnormality: Myocardial scar. 
